# Data on the scope of the literature on sustainable intensification 1997–2016: Bibliography, geography and practical approaches

**DOI:** 10.1016/j.dib.2018.06.029

**Published:** 2018-06-22

**Authors:** Meike Weltin, Ingo Zasada, Jana-Maria Plogmann, Fee-Nanett Trau, Annette Piorr

**Affiliations:** Leibniz-Centre of Agricultural Landscape Research, Eberswalder Str. 84, 15374 Müncheberg, Germany

**Keywords:** Systematic literature review, Classification, Agricultural systems, Food production

## Abstract

The data presented in this DiB article offers a full overview on the scientific sustainable intensification literature from 1997 to 2016. It consists of articles retrieved from the Scopus and the Web of Science databases that feature “Sustainable Intensification” (SI) as search term in title, abstract or author keywords. Information on bibliography, geographic focus and proposed sustainable intensification practices of each publication is recorded. The suggested sustainable intensification practices were assigned into 26 SI approaches constituting bundles of practices using a qualitative classification approach. The data is related to the research article entitled”Conceptualising fields of action for sustainable intensification. A systematic literature review and application to regional case studies” [1]. The information builds a baseline to assess the developments of the knowledge on SI and especially its practical implementation in depth. The database provides a comprehensive and structured overview of the SI literature and guidance for scholars and practitioners working on the topic.

**Specifications Table**

**Table:** 

Subject area	*Agricultural and Biological Sciences*
More specific subject area	*Sustainable intensification*
Type of data	*Table (Excel)*
How data was acquired	*Systematic search in Scopus and Web of Science databases*
Data format	*Raw and analysed*
Experimental factors	*The term “Sustainable Intensification” was searched in title, abstract and author keywords of all articles and review papers until December 31, 2016.*
Experimental features	*Bibliographic information on the selected articles was recorded as well as the geographic area of the study and sustainable intensification practices it primarily investigates.*
Data source location	*Global data*
Data accessibility	*With this article*

**Value of the data**

• The data provides a comprehensive overview on the two complete decades of scientific literature on sustainable intensification, including information on internal and external referencing.
• Targeted selection and assessment of relevant literature depending on specific keywords, geographical regions or sustainable intensification practices of interest is facilitated.
• The data builds a baseline for comparative as well as in-depth analyses of sustainable intensification implementation.
• Background information for studies of different disciplines related to sustainable intensification is summarized.

## 1 Data

The dataset provided with this article allows a comprehensive overview of the relevant scientific literature in the field of sustainable intensification (SI) research and was analysed in the systematic literature review by Weltin et al. [1]. Sustainable intensification is the umbrella term of a scientific discussion that seeks ways to ensure food production at less environmental cost [2], [3]. The number of included articles in the dataset is 349 covering the years 1997 to 2016. For each article bibliographic information is provided, such as authorship, title, year of publication, journal, author keywords, number of citations within and outside the analysed body of literature, and references. The geographic area(s) the article focuses on is additionally included, differencing world regions. The dataset further contains information on which of 26 identified SI approaches a publication mainly refers to.

## 2 Experimental design, materials and methods

The Scopus (https://www.scopus.com/) and the Web of Science (https://apps.webofknowledge.com/) databases were used to collect relevant articles. They represent the two main collections of academic literature [4]. For that purpose, the term “Sustainable Intensification” was searched for in title, abstract and author keywords of research articles and review papers. The final database consists of 349 articles representing all published articles up to December 31st, 2016. The overlap of the two databases is 271 articles. 59 are available in the Scopus database exclusively, 19 in the Web of Science respectively. Available bibliographic information of each article provided was retrieved. Citation records were retrieved from Scopus. The Web of Science was only used for the 19 articles exclusively registered there. Regarding citations, the number of citations of any given article by the other selected papers was tallied, representing the internal relevance of the article. Due to data formats, this was only done for the 330 articles originating from Scopus. The geographic focus area was extracted from the title or abstract of the paper.

The most important sustainable intensification practices were identified by evaluating the abstracts and conclusions of the articles. Following the approach by Gao et al. [5] an iterative multidisciplinary expert evaluation including economics, geography, natural resource management, agricultural sciences was applied to identify similar practices in order to cluster them into consistent bundles of practices forming SI approaches. Finally, 26 SI approaches were specified, to which the papers were assigned accordingly. Detailed information concerning the practices represented in approach is included in the metadata file of the provided dataset.
